# A Manual of Operative Surgery

**Published:** 1892-03

**Authors:** 


### Reviews of Books.

*A Manual of Operative Surgery.* By FREDERICK TREVES, F.R.C.S. Two Vols. Pp. 775 and 775. London: Cassell & Co. 1891.

We have already, in reviewing a work in these pages, expressed an opinion that no single man could write, unaided and from his own experience, a completely satisfactory work on Operative Surgery. If anything could make us modify the opinion then expressed, the work before us would do so. The author has brought to bear on his task indefatigable industry, accurate anatomical knowledge, varied practical experience, and a clear and often picturesque style; and he has given us a work of which he and the hospital to which he belongs may well be proud.

Looking into the details of the work, we have formed the most favourable opinion of the excellence, from the literary as well as practical standpoint, of Part I., dealing with general principles. Chapter II., on the operator, is a model of crisp and polished literary work, and is also brimful of clear, sound sense. No surgeon can read it without being the better for it. Part II., on the administration of anæsthetics, is written by Dr. Hewitt, and is a sound and sufficiently full piece of work. Of the rest of Vol. I., dealing with the ligature of arteries, operations on nerves, amputations, operations on the bones and joints, and tenotomy, we must speak in terms of high praise. On these subjects Mr. Treves is evidently at home; he writes with the double authority of full anatomical knowledge and extensive personal experience. The chapters on amputations are particularly good.

The second volume, although in some parts of special excellence, is here and there open to criticism. Naturally, the least satisfactory portions are those where personal experience is least. Thus in plastic surgery, a part well handled as a whole, the operation for ruptured perineum would scarcely satisfy the best surgery of the present day. If Mr. Treves had been able to bring his cultured surgical experience to bear on methods other than that described in Galabin's work, we are convinced that, in comparison with them, it would have been rejected. Part IX., on operations on the neck, is particularly good. On the subject of the tongue our author, in that operation in which he has had a most conspicuous success, is most modest. Preliminary ligature of the lingual arteries, a proceeding rather to be fought shy of by most surgeons, is, in Mr. Treves' hands, a mere anatomico-surgical exercise, or casual preliminary to the major or proper operation. For the majority of us it would be regarded as the most difficult part of the operation. Part IX., on abdominal operations, is, in many respects, excellent; in some, as in the important subject of intestinal obstruction, it is authoritative. We are glad to see that Mr. Treves has come to believe in enterotomy as a remedial measure of value in certain cases of obstruction. On the subject of hysterectomy for fibroids, in which it is evident our author has not had much experience, we cannot speak so favourably. Here he lets his zeal somewhat outrun his discretion. His love for the intra-peritoneal treatment of the pedicle—a love which all good surgeons share with him—leads him into a wholesale condemnation of the extra-peritoneal methods. No doubt we shall some day come to adopt intra-peritoneal treatment of the pedicle entirely; but so long as this method shows a mortality nearly double that of the extra-peritoneal, cautious surgeons will reserve the right to select their methods.

Operations on the intestines are most admirably described. We should have liked to have seen a description of Reclus' simple and excellent method of performing colotomy, an operation which has found much favour in the provinces, and which probably, in harmony with the history of other surgical improvements, will soon become known and appreciated in the metropolis.

Curiously enough, one page, and one page only, is devoted to the surgery of the eye; and that is, the destructive operation of excision of the eyeball. Might not a curious commentator on specialism here find a theme in the practice of the London Hospital?

We lay down the volumes fully confirmed in the belief that here we have one of the best single works on operative surgery ever written. If, here and there, we feel that we are in opposion to the author, we are conscious that the choice is between the better of two goods, rather than between good and bad. And, always, we know we shall return to these volumes for good advice and clear description in the difficult and trying art which it is our lot to cultivate.

